# Supplementary figures and images for: What’s behind the Dashboard? Intervention Mapping of a Mobility Outcomes Monitoring System for Rehabilitation
Source: Int J Environ Res Public Health. 2022 Oct 15;19(20):13303. doi: 10.3390/ijerph192013303 (PMC9602496; doi:10.3390/ijerph192013303)

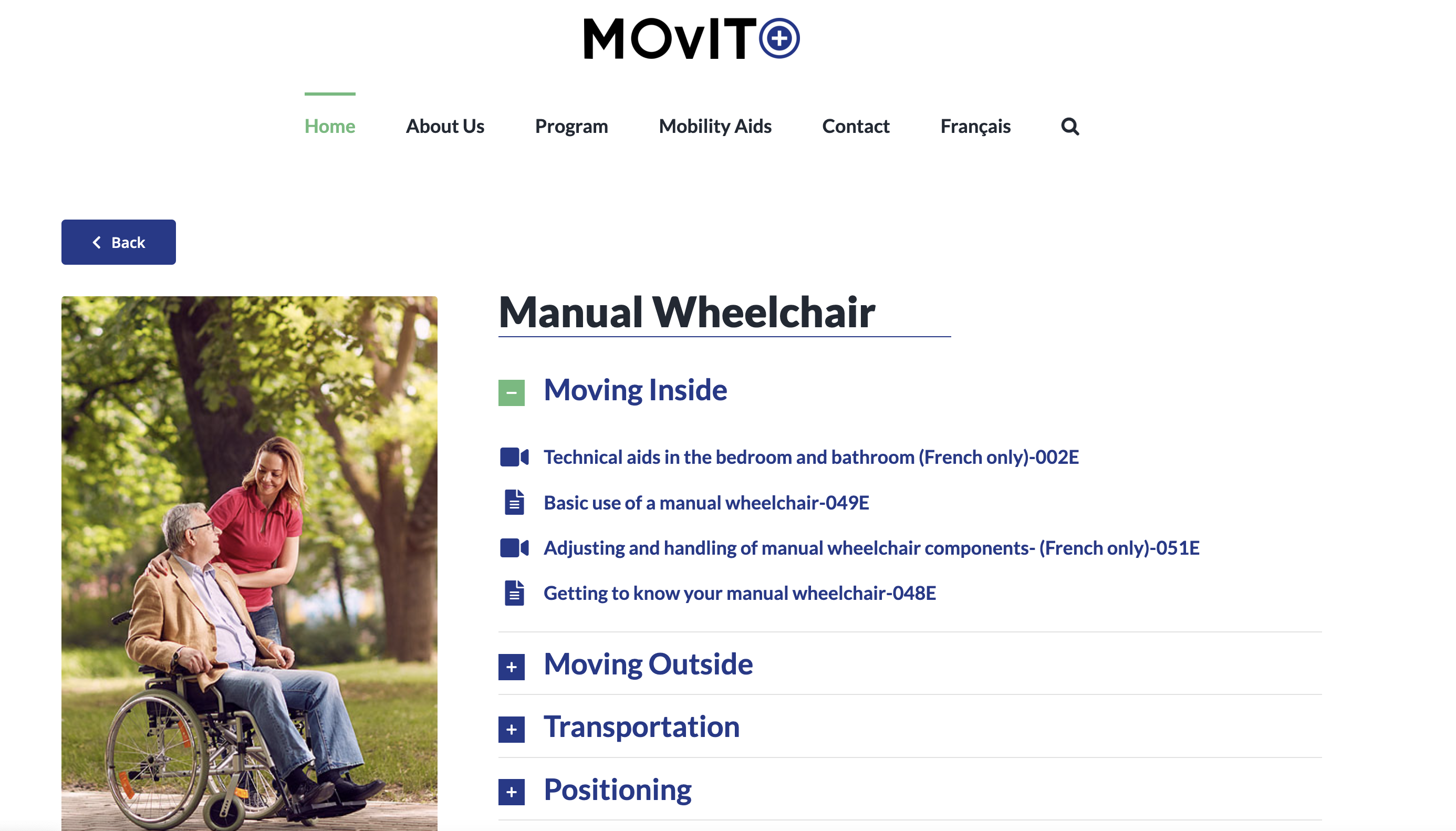

Supplement: Supplementary file 1 [file ijerph-19-13303-s001.zip › Supplementary material/Supplementary file 5_Figure S4.png]

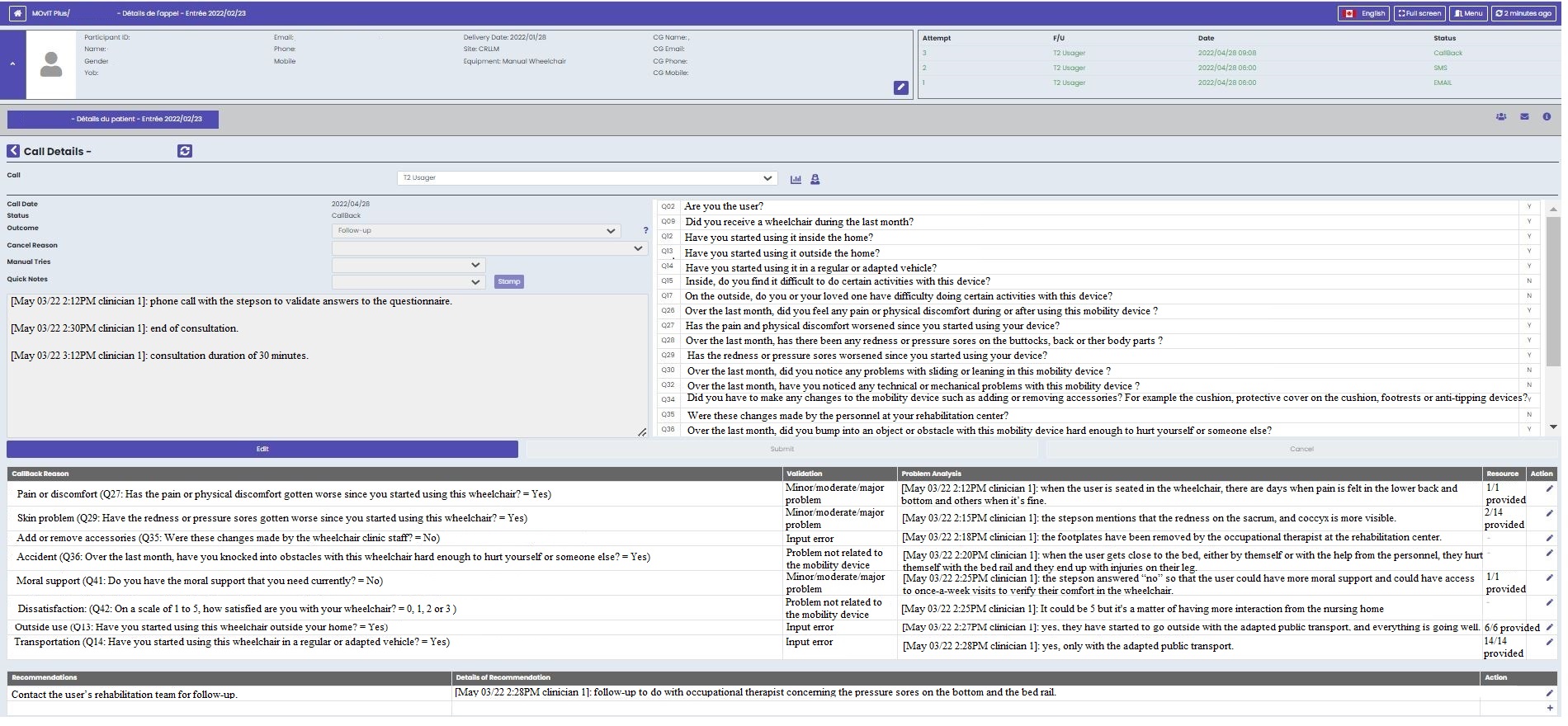

Supplement: Supplementary file 1 [file ijerph-19-13303-s001.zip › Supplementary material/Supplementary file 3_Figure S2.jpeg]

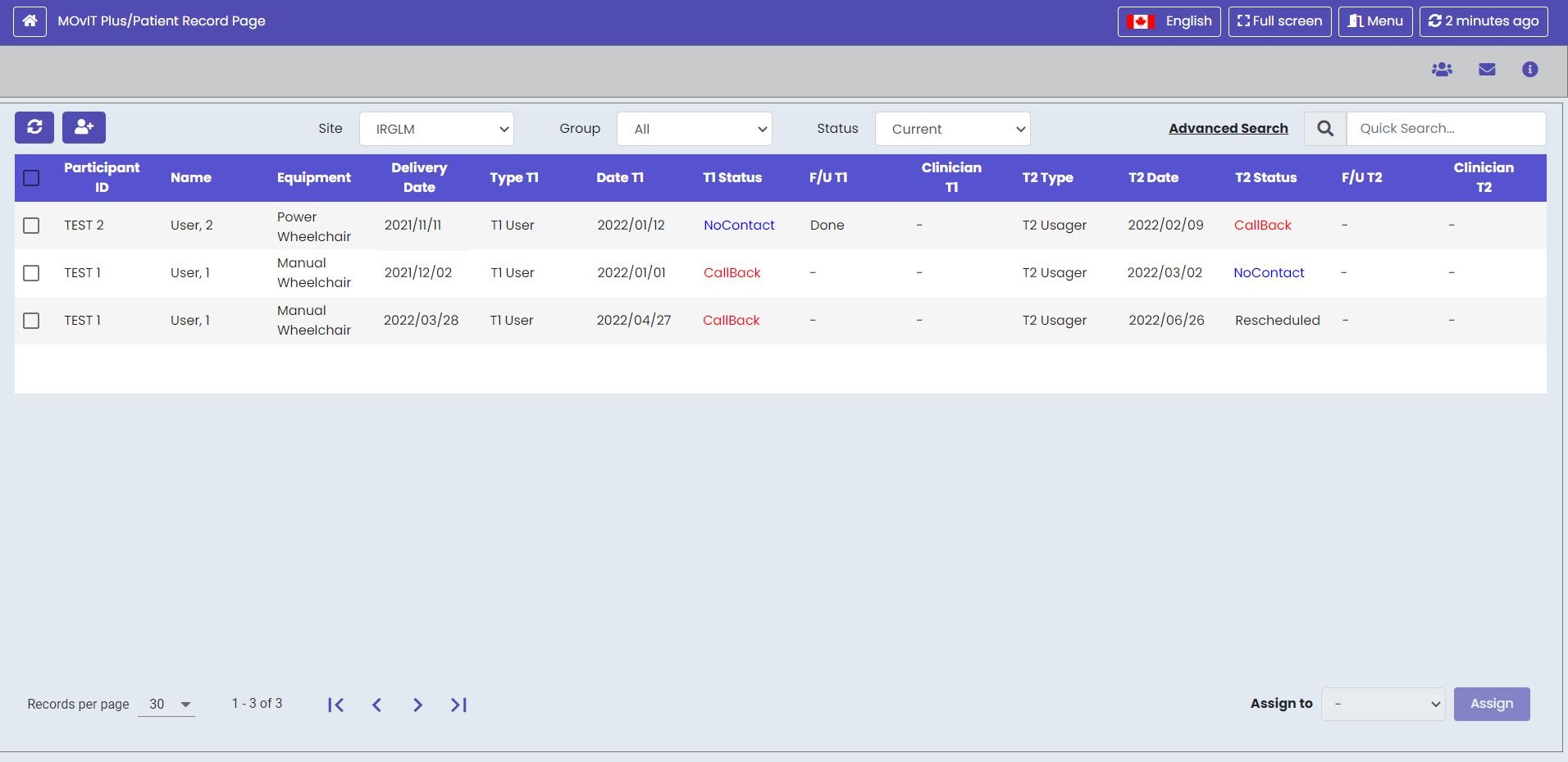

Supplement: Supplementary file 1 [file ijerph-19-13303-s001.zip › Supplementary material/Supplementary file 2_Figure S1.jpeg]

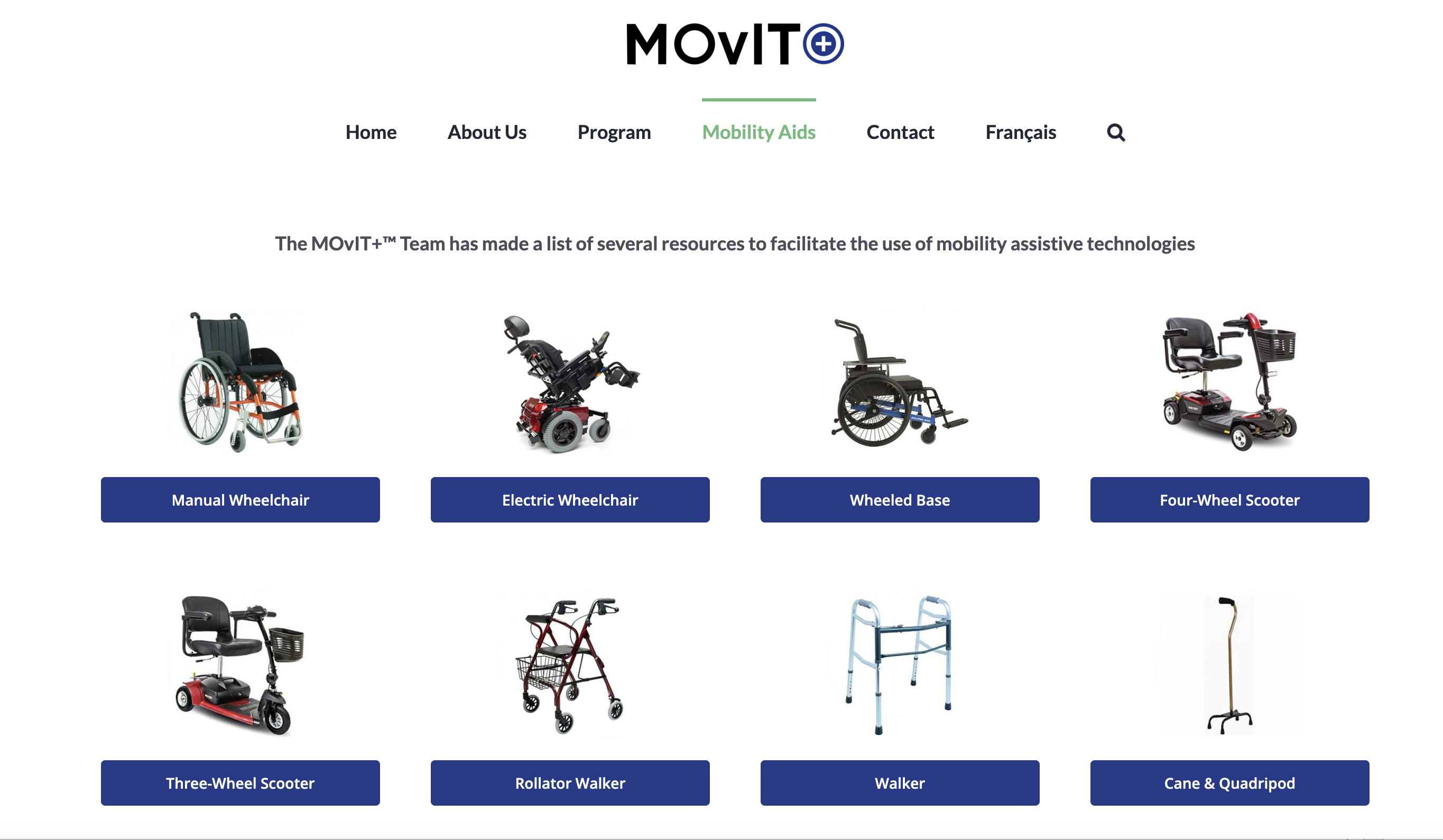

Supplement: Supplementary file 1 [file ijerph-19-13303-s001.zip › Supplementary material/Supplementary file 4_Figure S3.png]
